# Genetic Factors Modulate the Impact of Pubertal Androgen Excess on Insulin Sensitivity and Fertility

**DOI:** 10.1371/journal.pone.0079849

**Published:** 2013-11-20

**Authors:** Abigail R. Dowling, Laura B. Nedorezov, Xiaoliang Qiu, Joseph S. Marino, Jennifer W. Hill

**Affiliations:** 1 University of Toledo Medical Center, Center for Diabetes and Endocrine Research, Department of Physiology and Pharmacology, University of Toledo Medical Center, Toledo, Ohio, United States of America; 2 Dept. of Obstetrics-Gynecology, University of Toledo Medical Center, Toledo, Ohio, United States of America; 3 Department of Kinesiology, University of North Carolina, Charlotte, North Carolina, United States of America; John Hopkins University School of Medicine, United States of America

## Abstract

Polycystic ovary syndrome (PCOS) is the most common endocrine disorder of reproductive age women. The syndrome is caused by a combination of environmental influences and genetic predisposition. Despite extensive efforts, the heritable factors contributing to PCOS development are not fully understood. The objective of this study was to test the hypothesis that genetic background contributes to the development of a PCOS-like reproductive and metabolic phenotype in mice exposed to excess DHEA during the pubertal transition. We tested whether the PCOS phenotype would be more pronounced on the diabetes-prone C57BL/6 background than the previously used strain, BALB/cByJ. In addition, we examined strain-dependent upregulation of the expression of ovarian and extra-ovarian candidate genes implicated in human PCOS, genes containing known strain variants, and genes involved with steroidogenesis or insulin sensitivity. These studies show that there are significant strain-related differences in metabolic response to excess androgen exposure during puberty. Additionally, our results suggest the C57BL/6J strain provides a more robust and uniform experimental platform for PCOS research than the BALB/cByJ strain.

## Introduction

Polycystic ovary syndrome (PCOS) is a leading cause of female infertility, affecting 5–10% of reproductive-aged women [1], [2]. Common signs of PCOS include oligo- or anovulation, hyperandrogenemia, impaired follicle development in the ovary, and insulin resistance [3]. In humans, a clear genetic predisposition exists for the development of PCOS [4], [5], [6], as shown by studies of monozygotic twins and first-degree relatives of women with PCOS [7]. Despite extensive efforts, the genetic basis of PCOS is not fully elucidated. Studying the identity of and interactions among genes associated with PCOS may yield information about environmental influences on susceptibility and the pathophysiology of PCOS.

We reasoned that differences in genetic background might influence the development of a PCOS-like reproductive and metabolic state in mice. However, limited information exists regarding whether genetic factors contribute to susceptibility in mouse models of PCOS. Since most patients with PCOS begin to show symptoms at puberty and dehydroepiandrosterone (DHEA) is the first androgen to rise abruptly preceding puberty [8], [9], prepubertal administration of DHEA produces an animal model of PCOS with potential clinical relevance [10]. The administration of DHEA to immature female rats mimics PCOS by inducing cystic changes in the ovaries, precocious ovulation, acyclicity, and anovulation. As a result of such studies, a mouse model of PCOS was later developed using chronic prepubertal administration of DHEA in the BALB/cJ strain [11]. It is unclear whether this genetic background is required for DHEA induction of a PCOS-like state in mice, or whether this treatment can be applied to other, more commonly used mouse strains.

The purpose of this study was to assess the effect of background strain on the development of a PCOS-like reproductive and metabolic phenotype in mice. We hypothesized that the PCOS phenotype would be more pronounced on the diabetes-prone C57Bl/6 background, a strain susceptible to glucose intolerance, obesity, and diabetes [12], [13], [14]. We addressed four issues: 1) whether DHEA treatment triggers a PCOS-like reproductive and metabolic state in C57Bl/6J mice, 2) whether reproductive impairments are influenced by the genetic background, 3) whether DHEA treatment more effectively induces the metabolic traits associated with PCOS in C57BL/6J mice, and 4) whether candidate genes with strain-specific variants, association with PCOS, or involvement with steroidogenesis and gluconeogenesis are involved in phenotypic differences. Our studies suggest that significant strain-related differences exist in the metabolic response to DHEA treatment and that the C57BL/6J strain provides a more robust and uniform experimental platform for PCOS studies than the BALB/cByJ strain.

## Materials and Methods

### Animals and experimental protocol:

#### Ethics Statement

This study was carried out in strict accordance with the recommendations in the Guide for the Care and Use of Laboratory Animals by the National Institutes of Health. All procedures involving animals were approved by the institutional animal care and use committee (IACUC) at the University of Toledo, Health Science Campus.

A dehydroepiandrosterone (DHEA) administration protocol that has previously been published [11], [15], [16], [17] was used to induce a PCOS-like state in C57Bl6J (Stock # 000664) and BALB/cByJ (stock # 001026) mice (Jackson Laboratories, Bar Harbor, Maine). Briefly, 21-day-old female mice of the two strains were weaned into individual cages. At weaning, mice were alternately assigned to control and treated groups; no selection on the part of the investigators was involved. Thus, mice from each litter were evenly divided between the groups. No litter contributed more than three mice to a treatment group, and all groups contained mice from at least three litters. Two abnormally small (less than 8 g at 25 days) C57Bl/6 pups that came from large litters were excluded from the study. On day 25 of life, the mice were weighed, had submandibular blood drawn and then were given daily subcutaneous injections of DHEA (60 mg/kg body wt) (Sigma Aldrich, USA) in 0.08–0.12 mL of sesame oil or volume-matched sesame oil (control group) for 20 consecutive days. For each group, an N of 8–13 was used.

Mice were housed under controlled temperature (21°C) and illumination (12-h light, 12-h dark) and were given free access to water and Teklad 2016 global rodent diet (Harlan Laboratories, Madison, Wisconsin). Their food consumption and body weight were measured weekly from the start of injections, and the dose of DHEA was adjusted based on changes in weight each week. After 19 days of injections (day 44 of life), mice were fasted for 6 hours at the beginning of the light cycle, and a glucose tolerance test was performed with i.p. dextrose (2 g/kg body wt). Blood samples were taken from tail laceration and measured at 0 (fasting glucose), 15, 30, 45, 60, 90, and 120 minutes. Glucose levels were measured by the AlphaTrak mouse blood glucose monitoring system (Abbott Laboratories, North Chicago, IL). On the morning of day 46 of life and following an overnight fast, transcardial exsanguination under isoflurane anesthesia was performed to collect blood. Serum was separated by centrifugation at 4000 g for 10 min, aliquotted, and stored at −80°C. Freshly dissected ovaries were weighed and one ovary was processed for morphological studies and the second ovary was flash frozen for protein and RNA extraction. Gonadal fat depots and subcutaneous fat depots were weighed and compared between groups. Liver was flash frozen and stored at −80°C for protein and RNA extraction. Body composition was measured the morning of dissection by nuclear magnetic resonance (NMR) by Bruker Mini-spec (Bruker Optics, model mq 7.5 NMR Analyzer, Billerica, MA). Each mouse was scanned twice and both readings averaged to determine % fat, % lean, and % fluid of total body mass. Only mice in metestrus/diestrus were used for analysis of gene or protein expression, serum hormone levels, uterine weight, and the morphology of the ovary. To keep the age of testing constant and the number of mice sufficient for analysis, GTTs and body composition measurements were done irrespective of cycle stage.

### Estrous Cycle Analysis

From day 25 of life, each mouse was checked daily to observe vaginal opening. On the day of vaginal opening, each mouse began daily assays of vaginal cytology to determine each mouse's estrous cycle pattern. For 20 consecutive days, vaginal cells were collected via normal saline lavage daily between 11:00 and 13:00 hours and visualized under light microscopy. After first estrus was observed in each mouse, cytology was analyzed for percent time spent in each stage. Stages were assessed based on vaginal cytology as previously described [18]. Briefly, samples with primarily cornified epithelial cells indicated the estrus stage, primarily nucleated cells indicated the proestrus stage, both cornified cells and leukocytes indicated the metestrus stage, and primarily leukocytes indicated the diestrus stage. Their first day of estrus was determined by the first lavage in which cornified cells predominated.

### Serum Hormones

Fasting insulin levels were measured using the Ultra Sensitive Insulin ELISA (Crystal Chem Inc., Downers Grove, IL) with sensitivity to 0.1 ng/ml and intra and inter-assay variation of less than 10%. Serum estradiol and total serum testosterone were determined using Mouse/Rat ELISA kits (CalBiotech, Spring Valley, CA) with sensitivities of <3 pg/ml and intra- and inter-assay variances of 3.1% and 9.9%, respectively. All samples were run in duplicate and diluted to fit within the standard curve.

### Protein quantification

Tissues were homogenized in RIPA buffer (Millipore) containing protease and phosphatase inhibitors (Pierce, Thermo-Fisher) using the TissueLyser bead homogenizer (Qiagen). Homogenates were centrifuged and supernatants saved for protein analysis. Protein was quantified using a BCA protein assay (Pierce, Thermo-Fisher) according to the manufacturer's protocol.

### Real-time PCR

Total RNA from ovaries and liver was isolated using the Allprep DNA/RNA/Protein Mini Kit (Qiagen, CA). Total RNA from each sample were reverse transcribed to cDNA using the High Capacity Reverse Transcription Kit (Applied Biosystems). cDNA was analyzed by real time PCR using SYBR green technology (Applied Biosystems). Primer pairs can be found in Table S1. The reactions were run on a Step One system (Applied Biosytems) and quantified using the ΔΔCt method with GAPDH as the endogenous control gene [19].

### Western blot

Protein from ovary or liver was separated on 10% pre-cast gels (NuSep, Australia) and transferred to PVDF-FL membranes (Millipore). Membranes were blocked in Odyssey blocking buffer (Li-cor) diluted 1∶1 with tris-buffered saline (TBS) for 1 hour at room temperature. Following 2 washes with TBS-tween (TBS-T), membranes were incubated overnight at 4°C with primary antibodies specific for the insulin receptor beta subunit (Santa Cruz Biotechnology, Inc.), luteinizing hormone receptor (Santa Cruz Biotechnology, Inc), aromatase (Abcam), Cyp17a (Abcam), PCK1 (Abcam), and INHBB (Abcam) diluted 1∶1000 in blocking buffer. Following 2 washes with TBS-T, membranes were incubated for 1 hour at room temperature with species-appropriate secondary antibodies conjugated to an infrared dye (Li-cor) diluted 1∶5000 in blocking buffer. Proteins of interest were analyzed as a ratio of protein of interest divided by beta-actin (Sigma Aldrich) or alpha-tubulin (depending on size of protein of interest) total density in each lane. They were visualized and quantified using the Odyssey Scanning System (Li-cor). N = 4–7 per group for density quantifications.

### Ovarian Morphology

One ovary from each mouse was dissected and placed in 10% buffered formalin (Fisher Scientific) overnight, followed by 70% ethanol until embedded in paraffin and sliced. The slices were 10 um thick, and were imaged on the Nikon Eclipse 80i. Two sections per ovary were used and only follicles within which the ovum was visible were counted to avoid duplication. Numbers of corpus lutea, atretic follicles, preantral and antral follicles, primary and secondary follicles, and primordial follicles were counted.

### Statistical Analysis

Statistical analyses were carried out using GraphPad Prism 6 software (San Diego, CA). A two-way ANOVA was used when analyzing multiple groups with a Holm-Sidak multiple comparison test for intra-strain comparisons. If the response variable was found to depend on strain or treatment, stars are shown next to the bar legends. Multiple comparison results are shown above the bars. Student t-tests were used for comparisons between values of two groups. Area under the curve was calculated by GraphPad Prism using the trapezoid rule as the total area under the curve. Follicle count data were analyzed with a 2-way ANOVA. *P*<0.05 was considered statistically significant.

## Results

### DHEA Treatment Leads to Effective Elevation of Androgens

**Figure 1:**
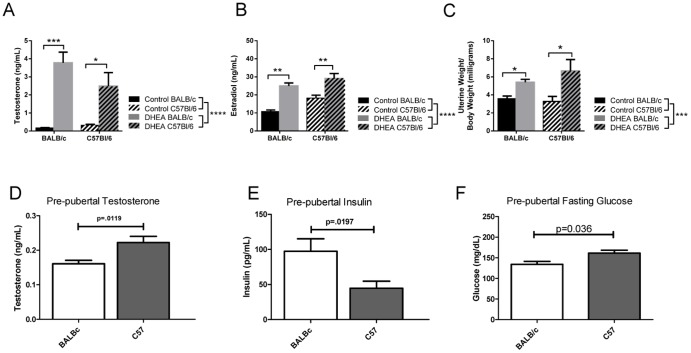
Hyperandrogenemia is induced by DHEA administration. A. Terminal serum testosterone levels. B. terminal serum estradiol levels C. Uterus weight compared to body weight upon dissection. D. Prepubertal testosterone levels. n = 8 E. Prepubertal fasting glucose. F. Prepubertal insulin levels. n = 7–9 for BALB/c groups and n = 9–10 in C57 groups. * means p<0.05, ** means p<0.01, *** means that p<0.001. ANOVA main effects are shown by the legend while the post hoc comparisons are over the column bars.

To confirm that DHEA treatments caused a hyperandrogenemic state, terminal serum was measured for levels of testosterone. As expected, treatment dramatically raised testosterone levels in both strains (p = 0.0001; Fig. 1A) [20]. Likewise, DHEA treatment greatly increased estradiol levels in both treated groups (p = 0.0001; Fig. 1B), which is likely the result of aromatization of testosterone via the canonical steroidogenic pathway [21]. As expected with elevated estradiol levels, DHEA treatment had a significant impact on uterus weight regardless of strain (p = 0.0002; Fig. 1C). Strain background had no impact on or interaction with any of these parameters. Given the likelihood of genetically-driven variation arising early in these two strains, we also examined their hormone levels prior to treatment. Glucose, insulin and testosterone levels at day 25 are shown in Figure 1D–F for both strains. At this early age, C57Bl/6 mice were found to have reduced insulin levels, higher glucose levels and, unlike in adulthood, modestly higher testosterone levels.

### DHEA Effects on Growth and Energy Storage

**Figure 2:**
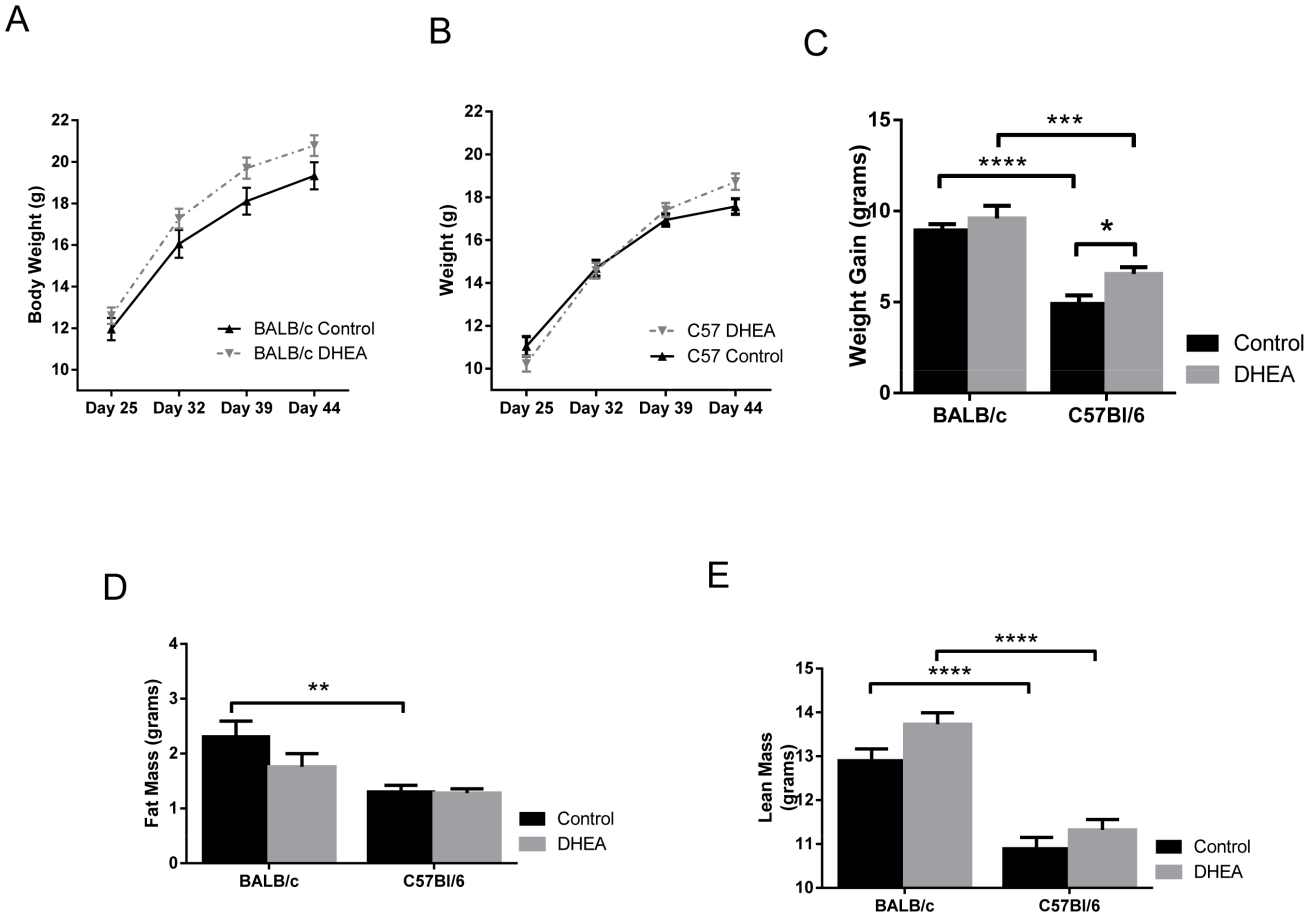
Body Composition of BALB/c and C57Bl6 strains following DHEA treatment. A. BALB/c and B. C57Bl/6 body weights over the course of treatment. C. Weight gain during study. D. Grams of fat mass by NMR (nuclear magnetic resonance). E. Grams of lean mass by NMR. N = 6–10 for all measurements.

DHEA increased the weight gained during the study, irrespective of strain (Fig. 2A–C, p = 0.0189). However, this effect was seen particularly in C57Bl/6 mice with multiple comparison analysis. In addition, while there is no altered fat mass (Fig. 2D), DHEA significantly increased lean mass overall (Fig. 2E, p = 0.0196). Food intake did not differ between treated and control groups (data not shown). Furthermore, strain had highly significant effects on weight gain (p<0.0001), fat mass (p = 0.0002), and lean mass (p<0.0001), which did not interact with treatment (Fig. 2A–E).

### Glucose Measurements and Tolerance

**Figure 3:**
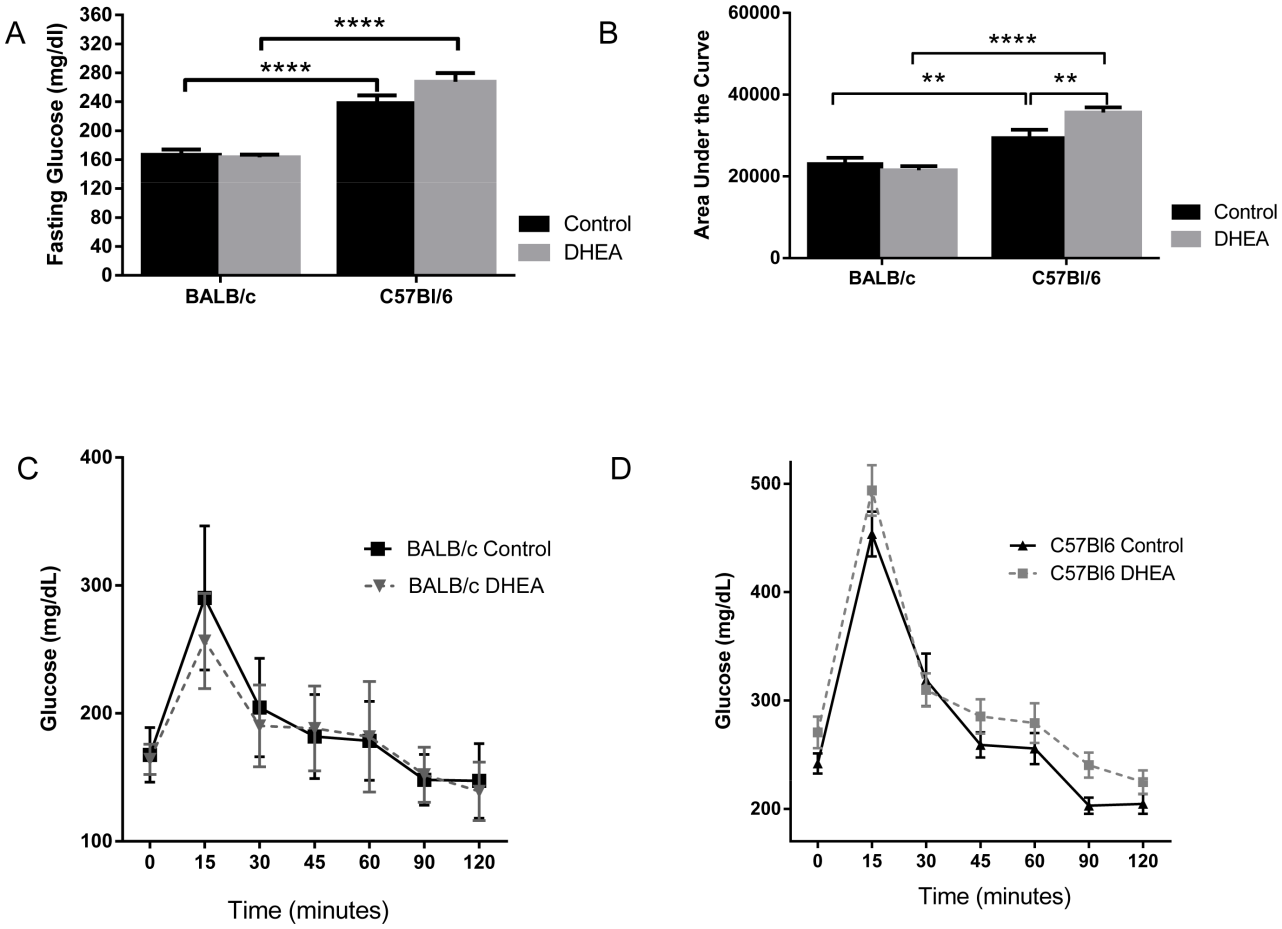
DHEA treatment impairs glucose tolerance in C57Bl6 female mice. A. Glucose tolerance test of BALB/cByJ females, treated and untreated. n = 7–9. B. Glucose tolerance test of C57Bl6 females, treated and untreated. n = 10. C. Fasting glucose levels of BALB/cByJ and C57Bl6 females. BALB/cByJ n = 9 and C57Bl6 n = 16 D. Area under the curve of glucose tolerance test of BALB/cByJ and C57Bl/6 females, n = 7–10. E. Fasting insulin levels upon dissection after overnight fast. n = 6–9.

The C57Bl/6 mice displayed fasting hyperglycemia in comparison to the BALB/c mice (p = 0.0001), but DHEA treatment did not alter fasting glucose (Fig. 3A). To further examine glucose regulation in these mice, we performed glucose tolerance tests on all groups (Fig. 3B–D). Importantly, an interaction was found between treatment and strain glucose tolerance, indicating that background strain influenced the response of the mice to the glucose bolus (p = 0.0106). Post analysis revealed that the C57Bl/6 groups were less glucose tolerant than their corresponding BALB/c groups with and without DHEA treatment. Interestingly, DHEA treatment slightly reduced glucose tolerance in C57BL/6 mice but not BALB/c mice (Fig. 3B, D). Insulin levels did not differ significantly between groups (Fig. 3E).

### DHEA Treatment Accelerates Puberty and Disrupts Estrous Cycle Length

**Figure 4:**
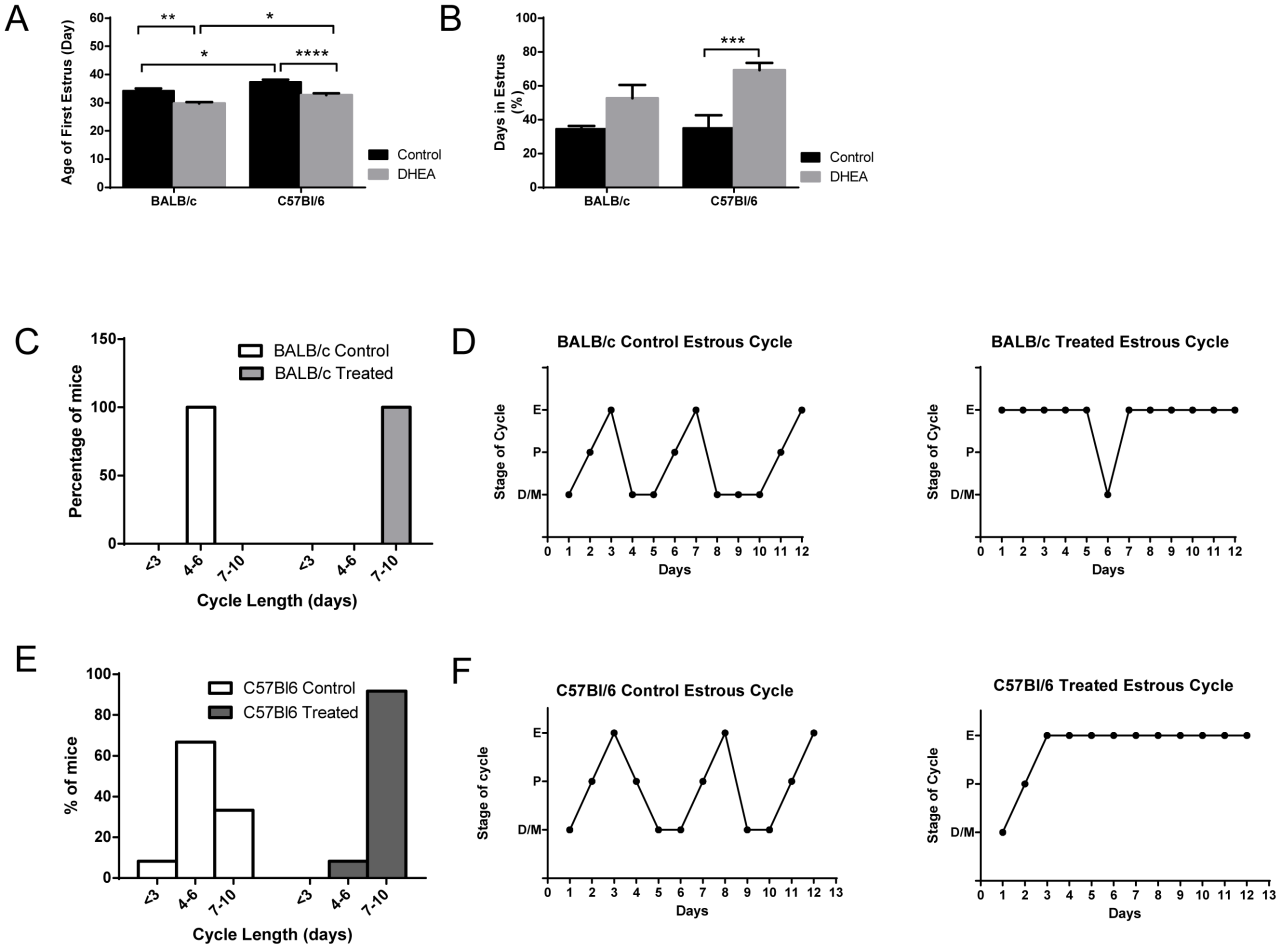
DHEA advances puberty and disrupts the estrous cycle in both strains. A. Day of life when first estrous smear was recorded. B. Percentage of days in estrus stage during the 20 day experiment. C. Average cycle lengths for BALB/cByJ females. n = 8 D. Representative estrous cycles of BALB/c females, untreated and treated. n = 6–8 E. Average cycle lengths for C57Bl6 females. n = 12–13 F. Representative estrous cycles of C57Bl6 females, untreated and treated.

No interaction was seen between strain and treatment in the timing of puberty. The initiation of estrous cycles occurred earlier in the BALB/c strain than the C57Bl/6 strain (p = 0.0002; Fig. 4A). DHEA advanced the first estrus day regardless of strain background (p<0.0001; Fig. 4A). Androgens in females are known to disrupt the estrous cycle [11]. We therefore examined estrous cyclicity in both strains of mice. Mice usually pass through a single day of estrus each cycle. DHEA treatment disrupted this pattern, extending estrus-like vaginal cytology for several days. This effect was present without accounting for strain (p = 0.0003), but was statistically significant in the comparison of C57Bl/6 control and treated groups (Fig. 4B). The average cycle length in both control groups was 4–6 days (Fig. 4C & E). The C57BL/6 strain exhibited more variability in estrous cycles as measured by cycle length; 100% of untreated mice fell in the 4–6 day range in the BALB/c strain, while 61.5% of C57BL/6 mice fell into this range (Fig. 4E). DHEA resulted in a lengthening of estrous cycles in both strain backgrounds, by an average of 4.5 days (data not shown). However, a more consistent disruption occurred in BALB/c cycles as evidenced by 100% of the animals exhibiting a cycle length exceeding 7 or more days after DHEA treatment (Fig. 4C & E), as opposed to 91.7% in the C57BL/6 strain.

### Ovarian Morphology

**Figure 5:**
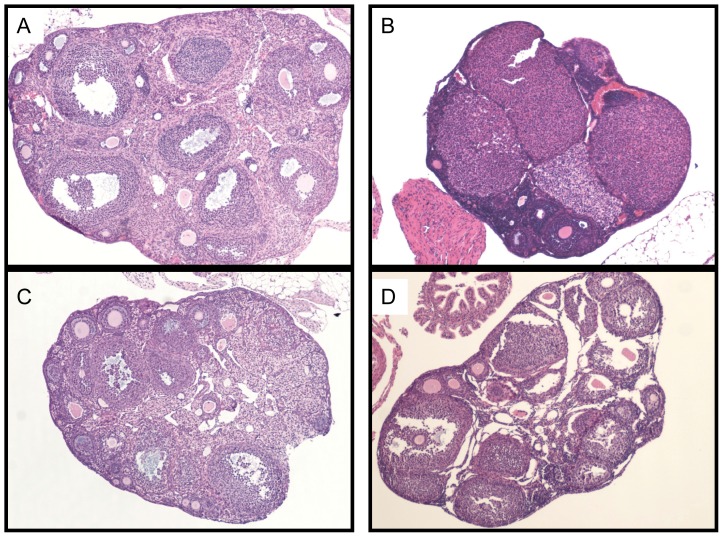
Ovarian Morphology. A) BALB/cByJ control ovary. B) BALB/cByJ treated ovary. C) C57Bl/6J control ovary. D) C57Bl/6 treated ovary.

Figure 5 shows a representative ovarian section from each group. The BALB/c and C57Bl/6 control ovaries show typical histology: large number of follicles in various stages of development, central medulla with stromal tissue and vasculature [11]. Ovarian weight was not significantly different between any of the four groups tested (data not shown). Comparison of follicle counts between groups showed more large follicles (antral and Graffian) in the BALB/c strain than the C57Bl/6 strain (Table 1). In addition, the number of primordial follicles was higher in BALB/c than C57BL/6 ovaries (Table 1).

**Table 1:** Ovarian follicular counts.

	BALB/c Control	BALB/c Treated	C57 Control	C57 Treated
**Primordial follicles**	17.25±1.830∧∧	15.55±1.681∧∧	10.63±1.822 #	8.0±5.686 #
**Preantral follicles**	5.625±0.565	3.727±0.6193	5.0±1.195	3.667±1.764
**Antral and Graffian follicles**	6.125±0.6391∧∧	4.545±0.4341∧∧	3.125±1.060 #	3.333±1.856 #
**Atretic follicles**	3.625±1.194	4.545±0.6923	7.250±1.46	5.0±2.082
**Corpora Lutea**	1.750±0.4532	2.364±0.5270	1.0±0.3273	1.0±0.5774

∧∧ marked groups are significantly different than the # marked groups. All significance marked in this table is p<0.05. n = 3–8.

### Strain Background Affects Gene Expression following DHEA Treatment

We next investigated whether genes with strain-specific variants (*Kit*), involvement with steroid hormone production (*Star*, *Cyp19a, Cyp17a1*), association with PCOS (*INHBB, Fem1b, Pgr*, Androgen receptor, *LHcgr*), or involvement with gluconeogenesis or insulin signaling (Liver *PepCk1*, Liver *IRβ*, ovary *IRβ*, Liver *G6Pase*) might be involved in phenotypic differences.

**Figure 6:**
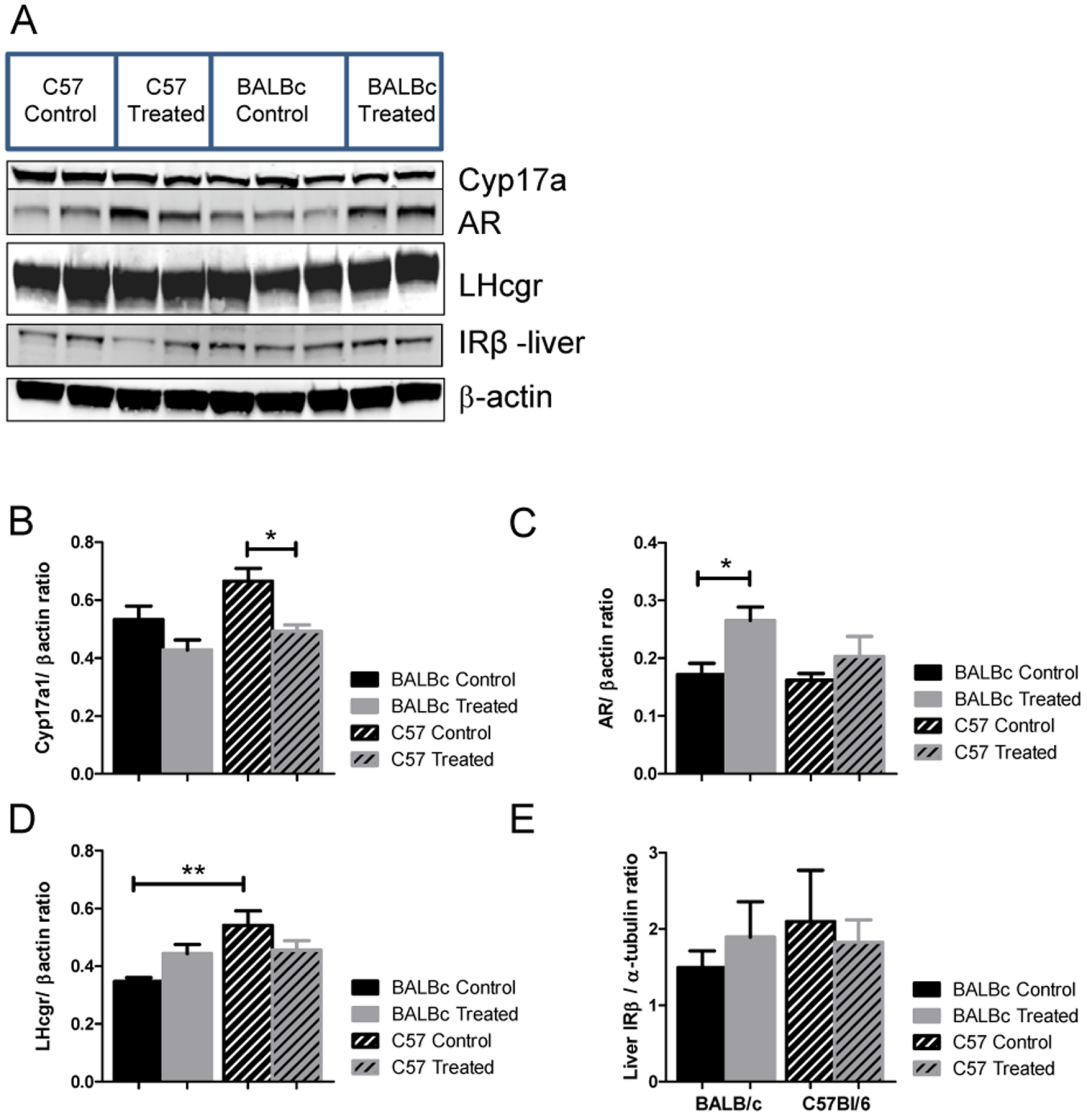
Western Blot densities. A. Representative western blots of target proteins and β-actin, the standard. At least two gels were used for density measurements, although only one gel is used for the figure. B. Cyp17a1/β-actin protein ratio. C. AR (androgen receptor)/β-actin protein ratio. D. LH receptor/β-actin ratio. E. Liver IRβ/α-tubulin ratio. For all westerns, n = 4–7 per group. For B–D protein was all from whole ovarian homogenate. E represents protein from whole liver homogenate.

First, ovarian mRNA from the two strains was analyzed for differences in expression of a subset of genes involved in steroidogenesis. *Cyp11* expression in the ovary, which encodes for the side-chain cleavage enzyme in the first step of steroidogenesis, showed no significant differences among the four groups. *Cyp17a1* encodes for the adrenal and gonadal enzymes 17α-hydroxylase and 17,20 lyase that convert progesterones into androgens. It showed no change in mRNA expression in response to DHEA treatment. However, its protein levels were significantly affected by both strain background (p = 0.0186) and DHEA treatment (p = 0.0024) without interaction between those factors. Specifically, C57Bl/6 mice responded to DHEA treatment with a suppression of Cyp17a product (Table 2 and Fig. 6A & B). Expression of steroidogenic acute regulatory protein (*StAR*), the cholesterol transport protein that controls the rate of steroidogenesis, was substantially lower in C57BL/6 ovaries but did not respond to treatment (Table 2). *Cyp19* encodes aromatase, the enzyme that converts androgens to estrogens. *Cyp19* gene expression was higher in BALB/c control mice than C57BL/6 control mice (Table 2). In both strains, *Cyp19* showed significant increases in response to androgen treatment (Table 2). This increase appeared to be larger in the BALB/c strain. However, no alteration in the protein levels of aromatase was seen (Fig. 6E). *Kit* showed no difference in ovarian expression among the groups (Table 2).

**Table 2:** Comparison of basal gene expression between strain backgrounds and treatments.

Gene Name	BALB/c Control vs. C57Bl/6 Control	BALB/c Control vs. Treated	C57Bl/6 Control vs. Treated
***Kit***	1.861±0.3267	0.9334±0.980	0.8515±0.0523
***Star***	0.1620±0.0887**	2.306±0.9229	1.432±0.3016
***Cyp17a***	0.7313±0.2591	0.7916±0.1957	1.342±0.2876
***Cyp19***	0.5343±0.1574*	33.28±3.256***	19.49±8.031**
***Fem1b***	1.538±0.1233**	1.344±0.172	1.218±0.127
***PGR***	0.6440±0.5702	1.650±0.4130	3.630±0.6332**
***LHcgr***	0.1470±0.0646***	3.578±1.765	0.7214±0.3027
***AR***	2.030±0.2006	1.176±0.1499	1.289±0.1205
***ERalpha***	1.449±0.3037	1.852±0.2234	1.476±0.4610
***Inhbb***	1.901±0.6666	1.331±0.2720	1.246±0.2633
***Liver PepCK1***	0.8149±0.0306	1.533±0.1845	1.343±0.1260
***Liver IR***	0.6440±0.5702	1.259±0.0883**	1.245±0.0831*
***IR***	1.693±0.1373***	1.852±0.301	1.476±0.1647
***Liver G6Pase***	0.9983±0.1914	1.264±0.2136	0.9735±0.1399

Expressed as mRNA fold change compared to the first listed group as reference group. All genes were assayed in whole ovary tissue unless otherwise noted. n = 5–8. Liver = hepatic gene expression.

* means p<0.05;

** means p<0.01;

*** means p<0.001.

We also examined a subset of genes known to be altered in the ovaries of women with PCOS [22], [23], [24], [25]. *Fem1b* was expressed at greater levels in C57BL/6 control mice but did not respond to DHEA treatment. We also found notable differences in the expression of several hormone receptors. *LHcgr* encodes for the luteinizing hormone receptor, which is prevalent in the ovary. The BALB/c strain expressed much less *LHcgr* mRNA than C57BL/6 mice under control conditions (Table 2). This difference was also seen at the protein level (Figure 6A & E). No clear response to DHEA treatment was seen, but a significant interaction between strain and DHEA treatment was present (p = 0.0205). In contrast, *PGR*, which encodes the progesterone receptor, was significantly different in the BALB/c strain compared to the C57 strain (Table 2). Finally, while androgen receptor gene expression was similar between the groups, DHEA treatment increased androgen receptor protein levels in general (p = 0.0078). Specifically, post analysis showed an increase in androgen receptor protein in the ovaries of BALB/c mice in response to DHEA (Fig. 6A & C and Table 2). There were no expression differences seen when *ERα* and *Inhbb* were analyzed.

Lastly, hepatic mRNA was analyzed for differences in expression of a subset of genes involved in glucose production, and insulin receptor beta subunit expression in the liver and ovary was measured. In the livers of control mice, mRNA and protein levels of *PepCK1*, an insulin-responsive gluconeogenic control gene, and the insulin receptor β subunit were similar in both strains regardless of treatment status. The *Pepck1* gene expression did respond to DHEA treatment (p = 0.0071) (Table 2). Hepatic *G6Pase* was similar among all groups, as shown in Table 2. In contrast, expression of hepatic *IRbeta* was significantly increased in both treatment groups in response to DHEA, although protein levels were unaffected (Fig. 6A & E). In the ovary, *IRbeta* mRNA was substantially higher in C57BL/6 mice, but did not significantly respond to DHEA treatment (p = 0.0608).

## Discussion

Chronic pre-pubertal administration of dehydroepiandrosterone (DHEA) in the BALB/cJ strain has been used to mimic PCOS [11]. BALB/cJ is an inbred mouse strain with low fecundity. Dorso-ventral vaginal septa consisting of a fibrous partition covered by epithelium have been reported in 38% of BALB/cJ females [26]. The fertility for females that retain this septum during mating is 14.3%, compared with 75.0% in normal females [27]. While the remainder of the reproductive tract is normal, higher embryo mortality and lower ovulation rates have also been reported for this strain [28]. It is unclear whether this genetic background is required for DHEA induction of a PCOS-like state in mice, or whether this treatment can be applied to other, more commonly used mouse strains.

C57BL/6J is the most widely used inbred strain and was the first to have its genome sequenced [29]. C57BL/6J mice exhibit glucose intolerance independent of obesity, reminiscent of human type 2 diabetes [30], [31]. Much of this glucose insensitivity results from impaired glucose-stimulated insulin release due to defective beta cell metabolism [32]. C57BL/6J mice also have a high susceptibility to diet-induced obesity. On a high-fat diet, these mice show higher weight gain and fat deposition per energy intake than other strains [33], [34], [35], resulting in insulin resistance, fasting hyperglycemia and diabetes [36], [37], [38], [39]. Finally, C57BL/6J mice fed an atherogenic diet develop atherosclerotic aortic lesions [40]. Indeed, the susceptibility of this strain to aortic lesions compared with other inbred strains led to the identification of key genes affecting atherosclerosis [41], [42].

Genetic background is known to play a large role in the phenotype of mouse models of metabolic disease. Indeed, *obese (ob)* leptin-deficient mice on the C57BL/6J background are infertile and initially obese, but then lose weight and die prematurely due to severe diabetes with islet β-cell failure [43], [44]. In comparison, *ob* mice on the BALB/cJ genetic background have more insulin resistance, but less white adipose tissue mass, and improved fertility [45]. Finally, *ob* mice on the FVB/N background have persistent hyperinsulinemia with hyperglycemia but without any signs of premature β-cell failure [46]. Therefore, modifier genes or allelic variants can substantially alter the reproductive and metabolic deficits seen on different mouse strain backgrounds.

Our findings confirm differences in body weight regulation, glucose tolerance, reproductive maturation, and adult reproductive characteristics in BALB/c and C57BL/6 mice in an untreated state. As expected based on the studies described above, at 25 days of age, C57BL/6 mice show reduced insulin levels and higher glucose levels. In addition, testosterone levels were modestly higher in this strain. This difference between strains was not present in adulthood and did not lead to any PCOS-like signs in untreated C57 mice. Nevertheless, it is possible that this early hormonal milieu may alter adult gene expression or glucose homeostasis. In adulthood, the BALB/c mice exhibited higher body weights from both increased adipose tissue and lean muscle mass, with earlier puberty and more regular 4–6 day estrous cycles in comparison to their C57BL/6 counterparts. They also displayed lower fasting glucose levels and better glucose tolerance than C57Bl/6 mice, as expected. We also found higher ovarian expression of *Star* and *Cyp19* in the Balb/c strain. *Star* encodes a cholesterol transport protein required for the production of steroid hormones, while the product of *Cyp19*, aromatase, catalyzes the conversion of C19 androgens to C18 estrogens. These findings suggest more steroidogenic capacity in the Balb/c strain. However, protein levels of aromatase were unchanged and basal levels of steroid hormones did not differ between the strains.

Our findings also confirm the PCOS-like reproductive phenotype found in other prepubertal DHEA-treated mice [11], in particular acyclicity and hyperandrogenemia (exogenously induced). On a gross level, DHEA treatment was effective in inducing this PCOS-like state in both strains. DHEA treatment also accelerated the pubertal transition. Interestingly, DHEA increased body weight primarily by increasing lean mass rather than adipose tissue. In addition, the expression of one ovarian gene was dramatically increased in both strains in response to DHEA treatment, namely *Cyp19*. Genetic variants of the aromatase gene have been associated with the development of PCOS [47], [48], [49]. Moreover, pharmacological inhibition of aromatase in rodents causes a PCOS-like metabolic phenotype that includes increased body weight, fat accumulation and insulin resistance [50]. Therefore, elevated aromatase expression could protect DHEA-treated mice from more extreme metabolic consequences of hyperandrogenemia. However, despite the observed upregulation of mRNA levels and the rise in circulating 17β estradiol following DHEA treatment in both strains, a corresponding increase in aromatase level of protein was not seen in the ovaries of these mice.

PCOS can involve, but does not require, the finding of a polycystic ovary. Indeed, 20% of women have polycystic ovaries, many without any other symptoms or evidence of impaired fertility [51], [52]. Morphologically, polycystic ovaries have normal or decreased primordial follicle numbers, while primary and preantral follicle counts are increased [53], [54], [55]. When ovulation is impaired, a preovulatory follicle or corpus luteum is absent [56]. However, the critical features for diagnosis of a polycystic ovary are increased volume, hyperplasia of the theca interna, an increase in the amount and density of stroma, thickening of the tunica, and increased numbers of antral follicles [57]. Normally, developmental arrest of a follicle leads to its atresia. However, antral follicles from polycystic ovaries are characterized by premature growth arrest without atresia [58], [59]. By the parameters we measured, DHEA treatment did not induce polycystic ovaries in either mouse strain.

Mouse models of PCOS frequently fail to exhibit polycystic ovaries as defined above, perhaps reflecting the multi-ovulatory nature of this species [60]. Nevertheless, other ovarian changes have been reported following androgen treatment of mice. In animals treated prepubertally for 90 days with dihydrotestosterone (a nonaromatizable androgen), van Houten and colleagues found abnormalities of follicle development, leading to atretic, fluid-filled structures in the ovaries of those mice [61]. Luchetti and colleagues found an increase in atretic follicles in the center of the ovary using a prepubertal 20 day DHEA treatment protocol [11]. They also found up to two large fluid-filled structures per ovary characterized by a thin layer of theca cells and a compacted formation of granulosa cells with no vascularized theca interna. In contrast, we found no change in the number of atretic follicles or the morphology of follicles following DHEA treatment.

The nature of the reproductive deficits following DHEA treatment differed between the strains. The BALB/c strain showed a more consistent increase in cycle length above 7 days, perhaps reflecting the low variability in estrous cycles in this strain in the absence of DHEA treatment. Androgen receptors in the ovaries of these mice also increased with DHEA treatment. Amplified transcriptional activity of *AR* is known to promote an androgenic intra-ovarian microenvironment, which may stimulate early follicular growth and contribute to mechanisms of follicular arrest found in PCOS [62]. Thus, the androgen receptor increase likely encouraged the lengthening of estrous cycles in these mice.

The majority of C57BL/6 mice have 4–6 day estrous cycles with clear cycle stages that allow them to serve as an appropriate strain for reproductive studies. However, they showed a wider variability in estrous cycle length compared to the BALB/c strain. In addition, a greater number of C57BL/6 mice exhibited a cessation of estrous cycles, defined as “cycles” longer than 10 days in length or vaginal cytology with a permanently cornified appearance. Several genes displaying higher basal expression levels compared to BALB/c mice may contribute to this phenotype. C57BL/6 mice exhibited increased ovarian insulin receptor expression that was not altered by DHEA treatment. Insulin directly affects steroidogenesis in granulosa and theca cells [63], [64], [65]. Increased insulin levels in synergy with LH may trigger premature LH receptor expression in small follicles leading to premature granulosa terminal differentiation [66], [67], [68], [69]. While DHEA did not increase LH receptors, the higher basal level of LH receptors seen in C57BL/6 mice (shown both by increased mRNA and protein levels) may contribute to the more extreme acyclicity following DHEA treatment in this strain. C57BL/6 mice also displayed higher basal ovarian Fem1b mRNA levels. While its ovarian function is unknown, Fem1b is expressed in human thecal cells [70] and Fem1b variants are associated with PCOS [71]. It should be noted that while the *Cyp17a1* mRNA expression was unaltered in the ovaries of both strains, Cyp17 protein levels fell in response to DHEA treatment, particularly in C57Bl/6 mice. Since the *Cyp17* enzyme converts progesterones to androgens, it is possible that its protein levels are subject to posttranslational negative feedback during DHEA treatment. At the same time, *PGR* levels in the ovaries of C57Bl/6 mice increased following treatment, suggesting sensitivity to progesterone is upregulated under these circumstances.

Importantly, the metabolic impact of DHEA treatment also differed based on the strain background. C57Bl/6 mice exhibited a significant increase in weight gain induced by DHEA treatment, along with reduced glucose tolerance. These metabolic impairments were absent in BALB/c mice following androgen administration. We found no evidence that this difference was due to altered hepatic gluconeogenesis or insulin sensitivity; indeed, hepatic insulin receptor gene transcription increased in both strains following treatment, although protein levels were unaltered. These results suggest that in C57BL/6 mice DHEA treatment is ineffective in inducing the type of hepatic insulin resistance seen in obese women with PCOS [72], [73]. Nevertheless, hyperandrogenemia may impair insulin sensitivity in muscle or white adipose tissue, as it does in PCOS patients [74], [75], [76], leading to the observed glucose intolerance.

Some limitations of the design of this study should be noted. By measuring gene and protein expression in whole ovaries, we may have missed variations at the level of specific cell types or follicle stages. In addition, pre-pubertal DHEA administration is one of many hormonal methods for inducing a PCOS-like state in mice [77], including prenatal T [78] or DHT [79], [80], postnatal T or testosterone propionate [81], [82], and pre-pubertal DHT [83]. All of these models share inherent limitations of using mice as a model of human reproductive function [60]. Nevertheless, these models allow fundamental research into the consequences of hyperandrogenemia for multiple tissues and are essential tools in PCOS research. However, surprisingly little attention has been given to the role of the strain in which these treatments have been evaluated. We have demonstrated that androgen-treated C57Bl/6 mice may be a better model for human PCOS because they develop both glucose intolerance and reproductive defects. Using a strain predisposed to the metabolic syndrome will permit further mechanistic study of the pathways that increase the risk for the metabolic and reproductive impairments associated with PCOS.

## Supporting Information

Table SI **RT PCR primers used for gene expression studies.**
(DOC)
