# Supplementary material for: Genetic Factors Modulate the Impact of Pubertal Androgen Excess on Insulin Sensitivity and Fertility
Source: PLoS One. 2013 Nov 20;8(11):e79849. doi: 10.1371/journal.pone.0079849 (PMC3835926; doi:10.1371/journal.pone.0079849)
Supplement: Table SI — RT PCR primers used for gene expression studies. (DOC) [file pone.0079849.s001.doc]

**Supplemental Table I: RT PCR primers used for gene expression studies.**

| **Gene Name** | **Primer name** | **Sequence** |
| --- | --- | --- |
| Androgen Receptor | *AR for* | CTGGGAAGGGTCTACCCAC |
|  | *AR rev* | GGTGCTATGTTAGCGGCCTC |
| Cytochrome P450, family 17, subfamily A, polypeptiode 1 | *Cyp17a1*  forward | AGTCAAAGACACCTAATGCCAAG |
|  | *Cyp17a1* rev | ACGTCTGGGGAGAAACGGT |
| Cytochrome P450, family 19, subfamily A, polypeptide 1 | *Cyp19a1* forward | GACAGGCACCTTGTGGAAAT |
|  | *Cyp19a1* reverse | CGGATAAGTAATGCCCCAGA |
| Glyceraldehydes-3-phosphate dehydrogenase | *Gapdh* forward | ATGTTTGTGATGGGTGTGAA |
|  | *Gapdh*  reverse | ATGCCAAAGTTGTCATGGAT |
| Glucose-6-phosphatase | *G6pase* forward | ATGTTTGTGATGGGTGTGAA |
|  | *G6pase* reverse | ATGCCAAAGTTGTCATGGAT |
| Inhibin, beta B | *INHBB* forward | AACTGCTCCCCTATGTCCTGG |
|  | *INHBB* reverse | CCGCTACGTTTCAGGTCCAC |
| Insulin Receptor, beta subunit | *InsR* RT sense | TCATGGATGGAGGCTATCTGG |
|  | *InsR* RT antisense | CCTTGAGCAGGTTGACGATTT |
| Proto-oncogene c-Kit | *Kit* forward | GCCTGACGTGCATTGATCC |
|  | *Kit* reverse | AGTGGCCTCGGCTTTTTCC |
| Luteinizing hormone receptor | *LHcgr* forward | GCCCTCCAGAGAAAAATTCAC |
|  | *LHcgr* reverse | CGACTGGTCAGGAGAACAAAG |
| Progesterone receptor | *Pgr*  forward | GCTTGCATGATCTTGTGAAACAGC |
|  | *Pgr* reverse | GGAAATTCCACAGCCAGTGTCC |
| Steroidogenic acute regulatory protein | *StAR* forward | GTGGCTGCCGAAGACAATC |
|  | *StAR* reverse | AGGTGGTTGGCGAACTCTATC |
